# Telomere length and prevalent chronic lung disease in middle-aged and older adults: socioeconomic heterogeneity and early-life tobacco exposure in the health and retirement study

**DOI:** 10.3389/fmed.2026.1954635

**Published:** 2026-09-30

**Authors:** Yazhe Dai, Haotian Cai, Wanyi Su, Shu Wang, Min Yang, Xiaolong Chuai

**Affiliations:** 1 Jilin Agricultural Science and Technology College, Jilin, China; 2 Gansu Agricultural University, Gansu, China

**Keywords:** cross-sectional association, health and retirement study (HRS), multivariable logistic regression, pulmonary diseases, telomere length

## Abstract

**Introduction:**

Telomere shortening is a hallmark of aging and age-related diseases, yet evidence linking it to pulmonary diseases remains limited.

**Methods:**

We included 4,994 participants aged ≥50 years from the 2008 HRS wave with available telomere measurement data. Pulmonary disease status was defined using the 2008 HRS self-reported physician-diagnosed chronic lung disease item, which includes conditions such as chronic bronchitis or emphysema and excludes asthma. Multivariable logistic regression models were used to examine the association between telomere length and the odds of prevalent chronic lung disease. Stratified analyses, restricted cubic spline (RCS) regression, and sensitivity analyses were conducted.

**Results:**

Each interquartile range (IQR) increase in telomere length was associated with a 14.3% lower odds of prevalent pulmonary disease (adjusted OR = 0.857, 95% CI: 0.774–0.945, *P* = 0.002). Participants in the longest telomere quartile (Q4) exhibited 38.0% lower odds compared to those in the shortest quartile (Q1). However, after excluding participants exposed to parental smoking during childhood, the association was attenuated and was no longer statistically significant (OR = 0.901, 95% CI: 0.736–1.091, *P* = 0.297).

**Discussion:**

Telomere shortening may be biologically related to pulmonary disease-related processes via cellular senescence and chronic inflammation. Shorter telomere length was associated with higher odds of prevalent self-reported chronic lung disease in the full sample; however, this association became non-significant after excluding participants exposed to parental smoking during childhood, indicating that the full-sample association should not be interpreted as independent of early-life tobacco exposure. Longitudinal studies are needed to clarify temporality.

## 1 Introduction

Telomeres—repetitive nucleotide sequences that cap chromosomal ends—progressively shorten with somatic cell division and are widely accepted as a biomarker of biological aging (1). Shortened telomere length has been implicated in multiple age-related conditions through mechanisms that include cellular senescence, impaired regenerative capacity, genomic instability, and sustained inflammatory signalling (2, 3). Epidemiological evidence links telomere attrition to cardiovascular disease, metabolic disorders, and diverse cancers, underscoring its potential relevance to chronic disease characterization and population-based association studies (4, 5).

Evidence specifically relating telomere dynamics to pulmonary disease is emerging but remains incomplete. Several clinical and translational studies report associations between shorter telomeres and chronic respiratory conditions such as chronic obstructive pulmonary disease (COPD) and idiopathic pulmonary fibrosis, suggesting that accelerated telomere erosion may contribute to alveolar epithelial cell senescence, defective repair, and fibrotic remodeling in the lung (6, 7). Other investigations, however, have produced inconsistent results or were limited by small sample sizes, narrow disease definitions, or restricted population sampling (8, 9). Moreover, heterogeneity in telomere measurement methods (tissue type and assay platform), adjustment for smoking and other environmental exposures, and limited control for early-life factors contribute to uncertainty in the literature (10).

Given the public-health burden of pulmonary diseases and the mechanistic plausibility linking telomere shortening to pulmonary pathophysiology, there is a need for large-scale, population-based analyses that use standardized telomere assays, comprehensive covariate adjustment, and exploration of effect modifiers. Using the nationally representative Health and Retirement Study (HRS), which includes validated salivary telomere measurements and extensive demographic, lifestyle, and childhood-environment covariates, the present study evaluates the association between telomere length and the odds of prevalent pulmonary disease in middle-aged and older adults and examines potential modifiers including sex, smoking history, and early-life exposures.

## 2 Materials and methods

### 2.1 Study population

This study utilized data from the US Health and Retirement Study (HRS) database. Initiated in 1992 by the University of Michigan with support from the National Institute on Aging (NIA) and the Social Security Administration (SSA), the HRS is a nationally representative longitudinal cohort study targeting Americans aged 50 years and older. The database employs a multi-stage stratified random sampling design, covering approximately 20,000 representative participants from 48 states and the District of Columbia. It encompasses multidimensional data on health status, economic conditions, family structure, social security, and healthcare utilization.

For the present analysis, we utilized the 2008 HRS wave (V 3.0). Participants aged ≥50 years with available leukocyte telomere length (LTL) measurement data were initially selected. After excluding individuals with missing data on sex, age, marital status, smoking status, alcohol consumption, body mass index (BMI), pulmonary disease status, race, socioeconomic status, or telomere outliers (defined as values beyond mean ± 3 standard deviations), a final analytical sample of 4,994 participants was included. The detailed participant selection process is illustrated in Figure 1.

**Figure 1:**
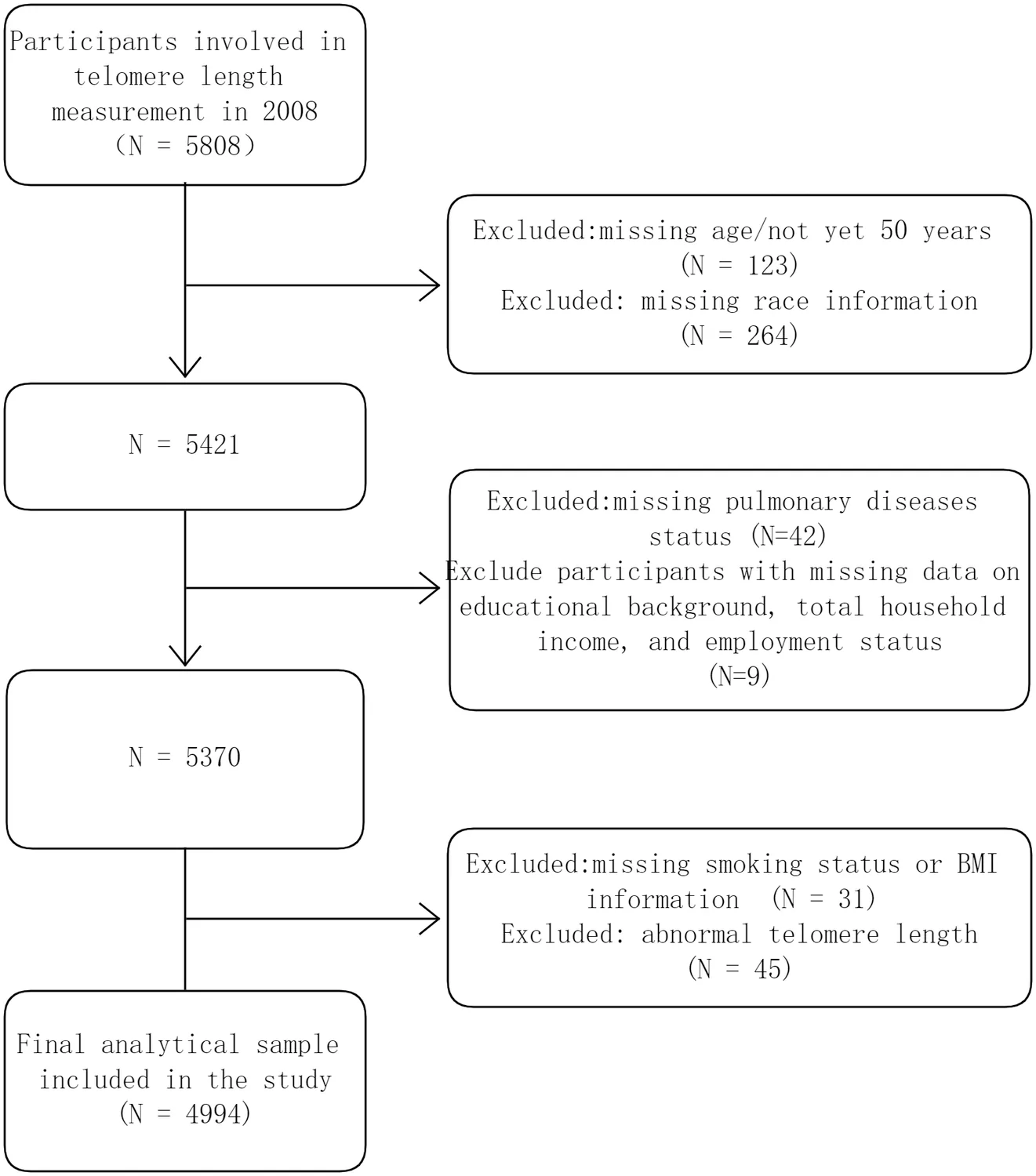
Flowchart of participant selection for the present study.

### 2.2 Telomere length data

Telomere length data from the 2008 HRS wave were used for this study. The 2008 telomere data release (Final Release, Version 1.0) included average telomere length data for 5,808 HRS respondents who consented and provided saliva samples during the 2008 interview wave. Average telomere length was determined using quantitative PCR (qPCR) by comparing the copy number of the telomeric repeat sequence (T) to the copy number of a single-copy gene (S) in each participant's sample (11). The resulting T/S ratio is proportional to the average telomere length.

### 2.3 Pulmonary disease definition

The Health and Retirement Study (HRS) is a long-term, nationally representative longitudinal project designed to investigate the health and economic status and transitions of the middle-aged and older adult population in the United States. This study utilized the HRS database to ascertain the prevalence of pulmonary disease within this population and conducted data analyses to examine the cross-sectional association between telomere length and prevalent pulmonary disease. Pulmonary disease status was derived from the 2008 HRS self-reported physician-diagnosed chronic lung disease item. Participants were classified as having pulmonary disease if they reported that a doctor had ever told them they had chronic lung disease, such as chronic bronchitis or emphysema; asthma was explicitly excluded according to the HRS questionnaire. Because the HRS core item does not provide subtype-specific diagnostic codes, such as COPD, IPF, acute infection, or neoplasm, disease-phenotype subgroup analyses could not be performed. Therefore, this outcome was analyzed as a single binary indicator of self-reported chronic lung disease.

### 2.4 Covariates

Demographic characteristics: Age (≥50 years, with secondary stratification at 75 years), sex (male, female), marital status (unmarried, married), race (white, black), and socioeconomic status (low status, high status).

Health-related behaviors: Smoking status (non-smoker, smoker), alcohol consumption (non-drinker, drinker).

Health status: Body Mass Index (BMI, calculated as weight (pounds) divided by height (inches) squared multiplied by 703, categorized as underweight, normal weight, overweight), self-rated health (excellent, very good, good, fair, poor), health insurance status (uninsured, insured).

Childhood adversity: Parental smoking during childhood (no, yes), childhood respiratory disease (no, yes).

### 2.5 Statistical analysis

Descriptive analysis was conducted by grouping participants according to pulmonary disease status. Categorical variables were described by number (n) and percentage (%). *P* values were used to assess group differences in covariates by pulmonary disease status, and chi-square tests were used for categorical variables.

To explore the association between telomere length and pulmonary disease, we employed multivariable logistic regression analysis, adjusting for potential confounding factors. The odds ratio (OR) and its 95% confidence interval (95% CI) were calculated to evaluate the association between telomere length and prevalent pulmonary disease. First, we calculated the interquartile range (IQR) of telomere length and grouped telomere length into quartiles (Q1-Q4) to assess associations across the telomere-length distribution. We fitted three logistic regression models. Model 1 was a univariable logistic regression model including telomere length only, without adjustment for other covariates. Model 2 adjusted for age, sex, marital status, race, socioeconomic status, and health insurance status. Model 3 further adjusted for smoking status, alcohol consumption, self-rated health, and BMI.

To assess potential selection related to missing childhood-exposure data, we compared telomere length and the prevalence of pulmonary disease between participants included in and excluded from the complete-case sensitivity analysis. The results of this missingness analysis are presented in Supplementary Table S2.

Model 1: A univariable logistic regression model including telomere length only, without adjustment for other covariates.

Model 2: Model 1 plus adjustment for age, sex, marital status, race, socioeconomic status, and health insurance status.

Model 3: Model 2 plus further adjustment for smoking status, alcohol consumption, self-rated health, and BMI.

Subsequently, we conducted stratified analyses. The odds ratio (OR) and its 95% confidence interval (95% CI) were calculated for each stratum to evaluate whether heterogeneity existed in the association between telomere length and prevalent pulmonary disease.

To verify the robustness of the results, we performed sensitivity analyses. Based on the fully adjusted model, we added two childhood variables: parental smoking during childhood and childhood respiratory disease. Participants with missing values for these two childhood variables were excluded from the complete-case sensitivity analysis (*n* = 1,239 excluded). We added both childhood parental smoking and childhood respiratory disease simultaneously to Model 3 (Model 4), excluded participants reporting childhood respiratory disease (Model 5a), and excluded participants exposed to parental smoking during childhood (Model 5b). We also conducted interaction analyses for childhood parental smoking and childhood respiratory disease separately (Model 6). In addition, to examine the impact of missing data on the results, we imputed the original missing data using multiple imputation methods and re-analyzed the data (Model 7).

All statistical analyses were performed using R software (version 4.4.2). A two-sided *P*-value < 0.05 was considered statistically significant.

## 3 Results

### 3.1 Descriptive statistics

A total of 4,994 study subjects were included for data analysis and grouped according to the presence or absence of pulmonary disease: no pulmonary disease and with pulmonary disease. Categorical variables were described by number (n) and percentage (%), with the results presented in Table 1.

**Table 1:** Categorical variables were described by number (n) and percentage (%).

Characteristics	Classification	No pulmonary disease(*n* = 4407)	With pulmonary disease(*n* = 587)	*P*
Sex (%)	Male	1874 (42.5%)	240 (40.9%)	0.478
	Female	2533 (57.5%)	347 (59.1%)	
Marital status (%)	Unmarried	1483 (33.7%)	239 (40.7%)	0.001
	Married	2924 (66.3%)	348 (59.3%)	
Smoking status (%)	Non-smoker	2046 (46.4%)	121 (20.6%)	<0.001
	Smoker	2361 (53.6%)	466 (79.4%)	
Alcohol consumption (%)	Non-drinker	2042 (46.3%)	319 (54.3%)	<0.001
	Drinker	2365 (53.7%)	268 (45.7%)	
BMI (%)	Underweight	49 (1.1%)	14 (2.4%)	0.029
	Normal weight	1308 (29.7%)	179 (30.5%)	
	Overweight	3050 (69.2%)	394 (67.1%)	
Self-rated health (%)	Poor	242 (5.5%)	108 (18.4%)	<0.001
	Fair	792 (18.0%)	190 (32.4%)	
	Good	1398 (31.7%)	204 (34.8%)	
	Very good	1474 (33.4%)	72 (12.3%)	
	Excellent	501 (11.4%)	13 (2.2%)	
Health insurance status (%)	Uninsured	1377 (31.2%)	126 (21.5%)	<0.001
	Insured	3030 (68.8%)	461 (78.5%)	
Age (%)	≤75	3198 (72.6%)	404 (68.8%)	0.064
	＞75	1209 (27.4%)	183 (31.2%)	
Race (%)	White	3804 (86.3%)	520 (88.6%)	0.147
	Black	603 (13.7%)	67 (11.4%)	
Socioeconomic status (%)	Low status	1767 (40.1%)	313 (53.3%)	<0.001
	High status	2640 (59.9%)	274 (46.7%)	
Telomere length (%)	Q1	1057 (24.0%)	192 (32.7%)	<0.001
	Q2	1096 (24.9%)	152 (25.9%)	
	Q3	1110 (25.2%)	138 (23.5%)	
	Q4	1144 (26.0%)	105 (17.9%)	

Among the 4,994 study subjects, 4,407 had no pulmonary disease and 587 had pulmonary disease. Compared to those without pulmonary disease, individuals with pulmonary disease were significantly more likely to be unmarried, be smokers, be non-drinkers, be underweight, rate their health as “poor”, have health insurance, and have low socioeconomic status (*P* < 0.05). Females constituted a larger proportion among those with pulmonary disease compared to males, although this difference did not reach statistical significance (*P* > 0.05).

The distribution of telomere length by quartile showed that the Q1 group (shortest telomeres) had the highest proportion of individuals with pulmonary disease (32.7%). This proportion progressively decreased with increasing telomere length quartiles (Q4 = 17.9%), suggesting an inverse association between telomere length and prevalent pulmonary disease.

### 3.2 Association analysis

The association between telomere length and the odds of prevalent pulmonary disease is shown in Table 2. For each interquartile range (IQR) increase in telomere length (IQR=0.362), the odds of prevalent pulmonary disease were 21.3% lower (OR = 0.787, 95% CI: 0.711–0.868, *P* < 0.001). After adjusting for all covariates, this association remained significant, with a 14.3% lower odds of prevalent pulmonary disease (adjusted OR = 0.857, 95% CI: 0.774–0.945, *P* = 0.002). The adjusted associations of all covariates included in the fully adjusted model with prevalent pulmonary disease are presented in Supplementary Table S1.

**Table 2:** The association between telomere length and the probability of having pulmonary disease.

Variable	Model 1 (univariable)		Model 2 (demographic-adjusted)		Model 3 (fully adjusted)	
	OR（95%CI）	P	OR（95%CI）	P	OR（95%CI）	P
Each interquartile range (IQR) increase in telomere length	0.787 (0.711–0.868)	<0.001	0.816 (0.737–0.900)	<0.001	0.857 (0.774–0.945)	0.002
Quartiles of telomere length						
Q1	Ref		Ref		Ref	
Q2	0.763 (0.607–0.959)	0.021	0.790 (0.626–0.995)	0.045	0.880 (0.690–1.122)	0.304
Q3	0.684 (0.541–0.864)	0.002	0.725 (0.571–0.919)	0.008	0.866 (0.674–1.112)	0.261
Q4	0.505 (0.392–0.649)	<0.001	0.551 (0.425–0.711)	<0.001	0.620 (0.473–0.810)	<0.001
*P* for trend		<0.001		<0.001		0.001

When analyzed by telomere length quartiles, compared to the Q1 group (shortest telomeres), the Q4 group (longest telomeres) exhibited 38.0% lower odds of prevalent pulmonary disease (OR = 0.620, 95% CI: 0.473–0.810, *P* < 0.001). The *P*-value for trend was 0.001 in the fully adjusted model. In contrast, Q2 and Q3 were not statistically significant after full adjustment, indicating that the inverse association was most evident for the highest telomere-length quartile and the continuous IQR estimate.

### 3.3 Restricted cubic spline (RCS) curve

To further explore the association between telomere length and the odds of prevalent pulmonary disease, telomere length was included as a continuous variable in the restricted cubic spline (RCS) model to generate a dose-response curve, as shown in Figure 2. The results demonstrated an inverse, approximately linear association between telomere length and the odds of prevalent pulmonary disease.

**Figure 2:**
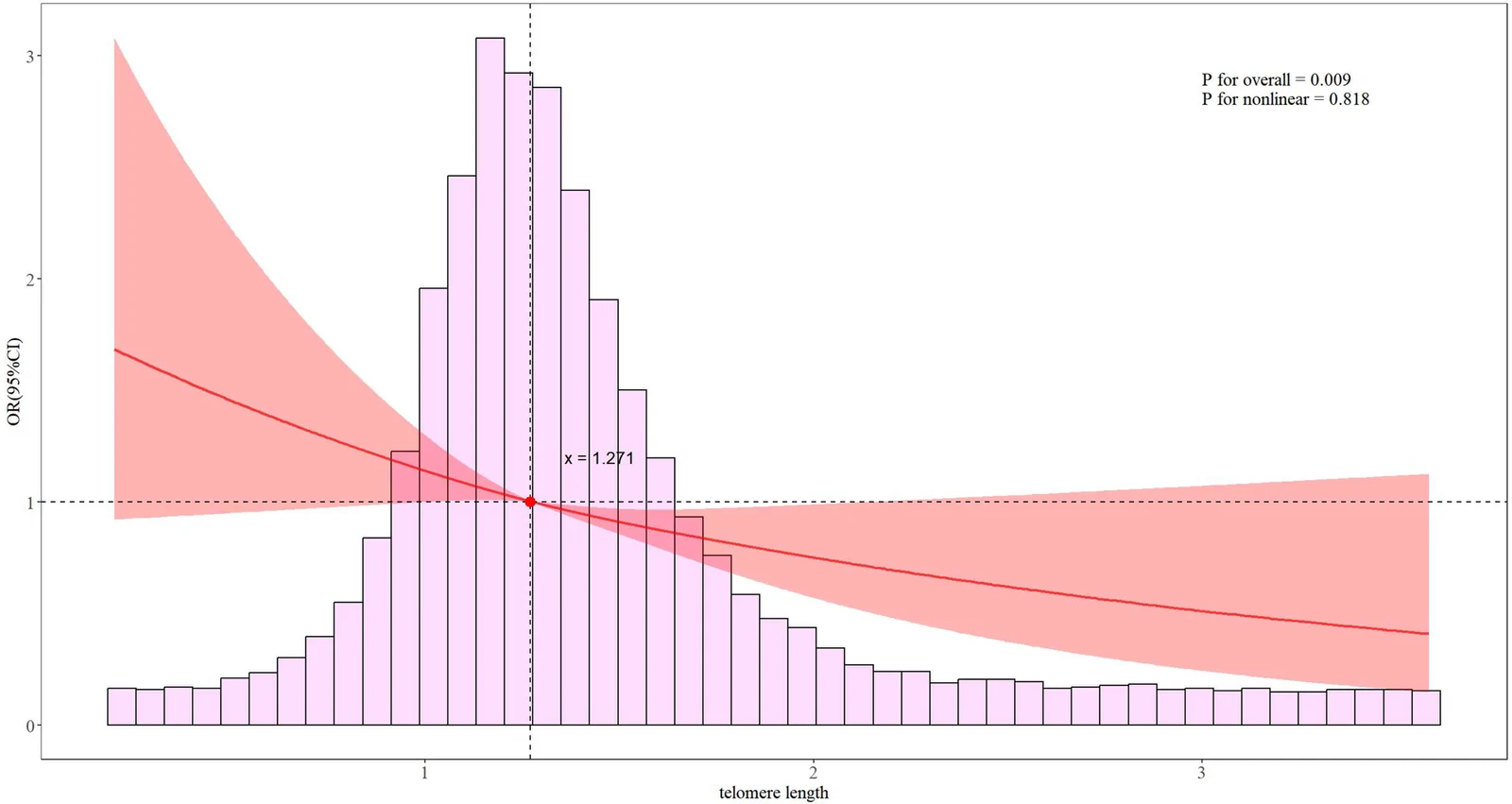
Cubic spline model analysis of the association between telomere length and the odds of prevalent pulmonary disease.

### 3.4 Stratified analysis

A weighted stratified logistic regression analysis was conducted to assess the association between telomere length and the odds of prevalent pulmonary disease across subgroups, with results visualized in a forest plot (Figure 3). Subgroup heterogeneity was observed for socioeconomic status (*P* for interaction = 0.033). Specifically, in the low socioeconomic status group, each interquartile range (IQR) increase in telomere length was associated with 5.2% lower odds of prevalent pulmonary disease (OR = 0.948), whereas the inverse association was stronger in the high socioeconomic status group (OR = 0.759). The interaction for sex did not reach statistical significance (*P* for interaction = 0.056), so sex-specific differences should be interpreted cautiously.

**Figure 3:**
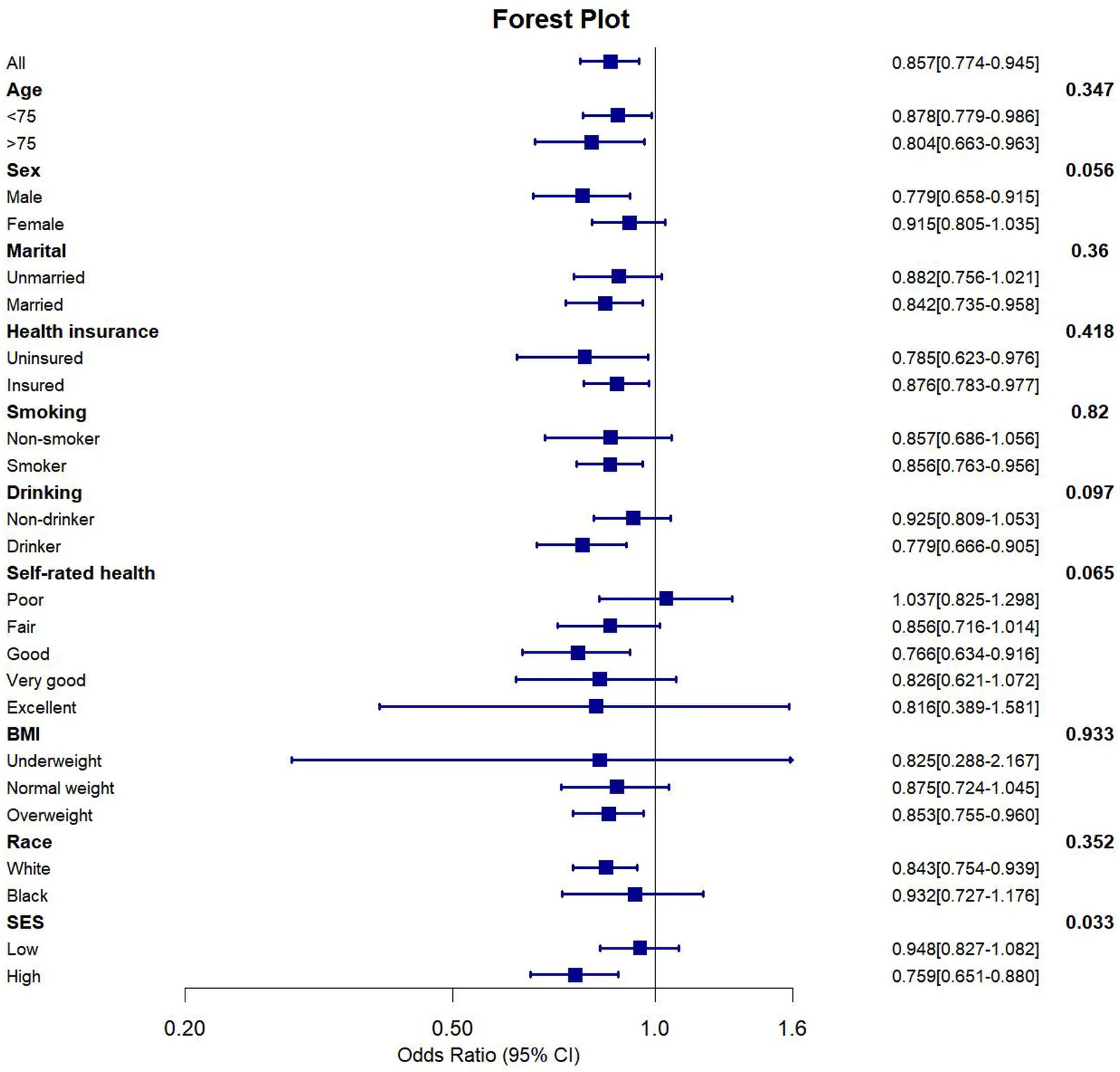
The association between telomere length and the odds of prevalent pulmonary disease.

### 3.5 Sensitivity analysis

After additionally adjusting for parental smoking during childhood and childhood respiratory disease, the inverse association between telomere length and prevalent pulmonary disease remained statistically significant (OR = 0.810, 95% CI: 0.719–0.909, *P* < 0.001). When participants with childhood respiratory disease were excluded, the association also persisted (OR = 0.806, 95% CI: 0.687–0.939, *P* = 0.007).

However, after excluding participants exposed to parental smoking during childhood, the association was attenuated and no longer statistically significant (OR = 0.901, 95% CI: 0.736–1.091, *P* = 0.297). This finding suggests that childhood parental smoking is an important confounding or effect-shaping factor. Therefore, the observed association between telomere length and prevalent pulmonary disease should not be interpreted as independent of early-life tobacco exposure.

We additionally compared participants included in the complete-case sensitivity analysis with those excluded because of missing childhood-exposure data (Supplementary Table S2). Mean telomere length was similar between the included and excluded groups (1.32 [SD 0.36] vs. 1.32 [SD 0.33], *P* = 0.883). However, the prevalence of pulmonary disease was higher among included participants than among those excluded because of missing data (12.7% vs. 8.8%, *P* < 0.001). These findings suggest that missingness was not associated with telomere length but may have been related to pulmonary disease status; therefore, potential selection bias due to complete-case exclusion cannot be entirely excluded.

Interaction analyses for childhood parental smoking and childhood respiratory disease did not show statistically significant interaction effects (*P* > 0.05). After multiple imputation of missing values and re-analysis, the results were directionally consistent with the primary complete-case analysis (Model 7), although this does not eliminate the possibility of selection bias related to missingness. Results are detailed in Table 3.

**Table 3:** Results of sensitivity analysis.

Model	OR(95%CI)	*P*
Model4		
Under the combined analysis of parental smoking during childhood and childhood respiratory disease	0.810 (0.719–0.909)	<0.001
Model5		
Excluding participants with childhood respiratory disease	0.806 (0.687–0.939)	0.007
Excluding participants exposed to parental smoking during childhood	0.901 (0.736–1.091)	0.297
Model6		
In the context of parental smoking during childhood	0.856 (0.675–1.090)	0.201
In the context of childhood respiratory disease	1.035 (0.819–1.305)	0.772
Model7		
Each interquartile range (IQR) increase in telomere length	0.873 (0.793–0.959)	0.005
Quartiles of telomere length		
Q1	Ref	
Q2	0.872 (0.690–1.101)	0.251
Q3	0.864 (0.679–1.099)	0.235
Q4	0.661 (0.513–0.850)	0.001

## 4 Discussion

Telomeres, repetitive nucleotide sequences at chromosomal termini, play a critical role in maintaining genomic stability (12) and cellular replicative capacity (13). Their progressive shortening is closely linked to aging and age-related biological processes, including pulmonary disorders (14). In the present cross-sectional analysis, shorter telomere length was associated with higher odds of prevalent self-reported chronic lung disease. Previous mechanistic and disease-specific studies have suggested that telomere dysfunction may be involved in chronic pulmonary conditions such as COPD and IPF (15, 16). However, because the HRS outcome used in this study is a self-reported physician-diagnosed chronic lung disease variable rather than a phenotype-specific diagnosis, these mechanisms should be interpreted as biological context rather than direct evidence explaining the composite outcome.

### 4.1 Biological plausibility and mechanistic insights

The observed inverse association between telomere length and prevalent chronic lung disease is biologically plausible, but it should be interpreted cautiously. Telomere shortening can trigger cellular senescence and inflammatory signaling, including senescence-associated secretory phenotypes, which may contribute to tissue remodeling in chronic disease contexts (15, 16). Oxidative stress, particularly related to smoking exposure, may also link telomere attrition with chronic lung injury in disease-specific settings (17). Nevertheless, these mechanisms primarily apply to COPD, IPF, or other defined chronic pulmonary phenotypes, whereas our HRS analysis could not distinguish specific pulmonary subtypes. Therefore, we revised the Discussion to avoid implying that these mechanisms directly explain all conditions included in the binary HRS chronic lung disease outcome.

Stratified analysis revealed socioeconomic heterogeneity in the association between telomere length and prevalent pulmonary disease (*P* for interaction = 0.033), with a stronger inverse association among participants with high socioeconomic status (OR = 0.759) than among those with low socioeconomic status (OR = 0.948). This difference may reflect the complex interplay between socioeconomic conditions, biological aging, and cumulative respiratory burden. Previous evidence suggests that socioeconomic disadvantage may be associated with accelerated biological aging, although associations between socioeconomic status and telomere length vary according to the socioeconomic indicator used, with relatively more consistent evidence for educational attainment (18). In older adults, higher educational attainment has been associated with longer leukocyte telomere length even after accounting for smoking, supporting the possibility that long-term socioeconomic conditions may become biologically embedded through behavioral, psychosocial, and inflammatory pathways (19). At the same time, socioeconomic disadvantage is independently associated with a greater burden of adverse respiratory outcomes. Population-based evidence from the United States has demonstrated persistent income- and education-related disparities in respiratory symptoms, COPD prevalence, and pulmonary function, which are only partly explained by differences in smoking (20). Lower socioeconomic status is also disproportionately associated with exposure to tobacco smoke, air pollution, occupational respiratory hazards, and barriers to high-quality health care (21). Against this background, the weaker telomere–pulmonary disease association observed in the low-socioeconomic-status group may reflect a greater contribution of multiple competing social and environmental determinants of pulmonary disease, thereby attenuating the relative contribution of telomere length. Conversely, among participants with higher socioeconomic status, a lower background burden of these adverse exposures may allow telomere length to more clearly reflect interindividual variation in biological aging associated with pulmonary disease. However, because socioeconomic status was categorized broadly in the present analysis and detailed information on lifetime income, occupational exposures, neighborhood environment, air pollution, and health-care access was unavailable, this interpretation remains exploratory and should be confirmed in prospective studies using multidimensional measures of socioeconomic position.

### 4.2 Early-life exposures and lifespan impact

A central finding of the sensitivity analyses was that the association between telomere length and prevalent pulmonary disease became non-significant after excluding participants exposed to parental smoking during childhood. This result substantially weakens any interpretation that telomere length is independently associated with pulmonary disease across all participants. Childhood secondhand smoke exposure may represent an upstream factor linked to both accelerated telomere shortening and later-life chronic lung vulnerability. Therefore, the primary association observed in the full sample may be partly driven or strongly modified by early-life tobacco exposure. Because this study is cross-sectional, we cannot determine whether telomere shortening precedes pulmonary disease, whether pulmonary disease-related inflammation accelerates telomere attrition, or whether both reflect shared early-life exposures. We therefore interpret parental smoking during childhood as a key context for understanding the telomere–pulmonary disease association.

This aligns with evidence that prenatal or childhood tobacco exposure may be associated with accelerated telomere shortening through epigenetic modifications, including telomere-region DNA methylation (22, 23). Early respiratory infections may also contribute to later-life pulmonary vulnerability. In disease-specific clinical contexts, individuals with telomere-related gene variants, such as TERT and TERC, can exhibit susceptibility to idiopathic pulmonary fibrosis and emphysema, particularly when exposed to smoking (24, 25). These observations provide biological context but should not be interpreted as proof of causality in the present cross-sectional HRS analysis.

### 4.3 Clinical interpretation and research implications

Given the cross-sectional design and the composite, self-reported nature of the chronic lung disease outcome, the clinical implications of our findings should be interpreted cautiously. Our results do not support using telomere length to guide monitoring intensity, initiate early intervention, or recommend specific pharmacologic treatments. Previous literature on telomere dysfunction, telomeropathies, antifibrotic or telomere-related therapeutic development, lifestyle factors, and senescence biology remains relevant as background (14, 26–30). However, these studies should not be interpreted as evidence supporting treatment recommendations from the present analysis. Disease-specific therapeutic implications require clearly defined pulmonary diagnoses, prospective validation, and treatment-response data, none of which were available in this study.

### 4.4 Limitations and future directions

This study has limitations. The cross-sectional design precludes causal inference; longitudinal studies are needed to establish temporality. Telomere length was measured in peripheral blood leukocytes, which may not fully reflect lung-tissue-specific dynamics. Future research should incorporate disease-specific pulmonary phenotyping, objective measures such as spirometry and imaging, and, where appropriate, advanced telomere assessment methods such as single telomere length analysis (STELA) and telomere-related gene evaluation in clinically relevant subgroups (31, 32). Because the sex interaction was not statistically significant in the revised analysis, we did not interpret sex-related mechanisms in the present study.

An important limitation is the composite and self-reported nature of pulmonary disease ascertainment. Although the HRS item primarily captures physician-diagnosed chronic lung disease such as chronic bronchitis or emphysema and excludes asthma, it does not distinguish specific pulmonary phenotypes, including COPD subtypes, IPF, acute infections, or neoplasms. Because telomere attrition is more directly linked mechanistically to chronic lung remodeling processes such as COPD and pulmonary fibrosis than to all pulmonary conditions, grouping heterogeneous diseases into a single binary outcome may dilute or obscure disease-specific associations. Thus, our findings should be interpreted as reflecting an association with self-reported chronic lung disease rather than with any specific pulmonary phenotype. Future studies incorporating ICD codes, spirometry, imaging, and subtype-specific diagnoses are needed.

Participants excluded because of missing childhood-exposure data had a lower prevalence of pulmonary disease than those included in the complete-case sensitivity analysis, although their mean telomere lengths were similar. Therefore, selection related to missing childhood data cannot be entirely excluded. Nevertheless, the broadly consistent findings obtained after multiple imputation provide some reassurance regarding the robustness of the primary association.

## 5 Conclusions

Shorter telomere length was associated with higher odds of prevalent pulmonary disease in middle-aged and older adults after adjustment for demographic, lifestyle, and health-related factors. Each interquartile range (IQR) increase in telomere length was associated with 14.3% lower odds of prevalent pulmonary disease in the fully adjusted model, and participants in the longest telomere quartile had 38.0% lower odds than those in the shortest quartile. However, after excluding participants exposed to parental smoking during childhood, the association was attenuated and was no longer statistically significant (OR = 0.901, 95% CI: 0.736–1.091, *P* = 0.297). This sensitivity finding suggests that the association observed in the full sample should not be interpreted as independent of childhood parental-smoking exposure and that early-life tobacco exposure is an important factor in interpreting the observed association.

Socioeconomic status, rather than sex, showed statistically significant heterogeneity in the revised stratified analysis. The inverse association was stronger among participants with high socioeconomic status than among those with low socioeconomic status, but this finding remains exploratory because socioeconomic status was measured broadly and detailed information on lifetime environmental and occupational exposures was unavailable.

Overall, telomere length may serve as a biological marker for studying cross-sectional differences in prevalent self-reported chronic lung disease, but it should not be used to infer future pulmonary disease onset or to guide clinical intervention based on the present data. Future research should prioritize longitudinal designs, objective pulmonary phenotyping, tissue-specific telomere measurements, and careful assessment of early-life environmental exposures.

## Data Availability

The original contributions presented in the study are included in the article/supplementary material, further inquiries can be directed to the corresponding authors.
